# *Brucella anthropi* Endocarditis: An Unusual Pathogen

**DOI:** 10.3390/idr18020032

**Published:** 2026-04-08

**Authors:** Fernando Baires, Erin Arias, María José Díaz, Cesar Burgos, Carlos A. Umaña Mejia, Justice Cruz, Joanne Cordero Guerra, Helen Hoffman, Jack Bordovsky, Jana Radwanski, Miguel Sierra-Hoffman, Amy C. Madril

**Affiliations:** 1 Facultad de Ciencias Medicas, Universidad Nacional Autónoma de Honduras, Tegucigalpa 11101, Honduras; ferbaires93@hotmail.com; 2 Facultad de Ciencias de la Salud, Universidad Latina de Costa Rica Powered by Arizona State University, San Jose 11501, Costa Rica; erinarias11@yahoo.com (E.A.); hellen.sierra@ulatina.net (H.H.); 3 Facultad de Ciencias Médicas, Universidad de Ciencias Médicas, San Jose 10108, Costa Rica; mariajose.diaz@ucimed.ac.cr; 4 Instituto Nacional Cardiopulmonar El Tórax, Tegucigalpa 11101, Honduras; cesar.burgos@unah.hn; 5 Facultad de Medicina, Universidad Autónoma de Guadalajara, Guadalajara 45129, Mexico; carlos.umana@edu.uag.mx; 6 Texas A&M University-Victoria Campus, Texas A&M University, Victoria, TX 77901, USA; leonardj@tamuv.edu; 7 Infectious Diseases and Pulmonary, Infectious Disease and Pulmonary Consultants, Victoria, TX 77901, USA; joanne.corderoguerra@gmail.com; 8 Medical Student, Alabama College of Osteopathic Medicine, Dothan, AL 36603, USA; jackbordovsky@gmail.com; 9 Citizens Medical Center, Victoria, TX 77901, USA; pharmerrad@gmail.com; 10 Infectious Disease and Internal Medicine, Sam Houston State University, Conroe, TX 77304, USA; 11 Department of Hospital Medicine, El Campo Memorial Hospital, El Campo, TX 77437, USA; amycmadril@gmail.com

**Keywords:** *Brucella anthropi*, *Ochrobactrum anthropi*, *Brucella*, acute endocarditis, taxonomy

## Abstract

Background: The genus *Brucella* has expanded considerably in the 21st century. With the advent of advanced phylogenetic analyses, a close genetic relationship between *Brucella* and *Ochrobactrum* has been identified, leading to reclassification of *Ochrobactrum* species within the genus *Brucella*. Among these, *Brucella anthropi* (formerly *Ochrobactrum anthropi*) is increasingly recognized as a rare cause of invasive human infection. We report a clinically significant case of *B. anthropi* infective endocarditis and review the available literature. Methods: We report a case of *B. anthropi* infective endocarditis and conducted a narrative review of the English-language medical literature through 2025. Cases were analyzed for demographics, clinical presentation, antimicrobial susceptibility, and outcomes. Results: A 75-year-old man with a prosthetic aortic valve and prior endocarditis presented with fever of unknown origin, weight loss, and prior transient ischemic attacks. Blood cultures grew *B. anthropi* after prolonged incubation. Transesophageal echocardiography demonstrated vegetations involving both the aortic and tricuspid valves, and the patient required targeted combination antimicrobial therapy due to persistent bacteremia. Seven additional cases of *B. anthropi* infective endocarditis were identified on review of the literature. Most patients had underlying valvular disease or prosthetic material. Reported lethality approached 25%. Antimicrobial susceptibility patterns were variable, underscoring the importance of targeted individualized therapy. Conclusion: Consistent with other Gram-negative bacilli, *B. anthropi* is a rare but established cause of acute bacterial endocarditis. Despite its rarity, it may represent an under-recognized cause of invasive disease. This case highlights the importance of prolonged culture incubation, careful microbiologic interpretation, and susceptibility-guided therapy.

## 1. Introduction

Recent advances in whole genome sequencing have prompted important taxonomic revisions within the family *Brucellaceae.* In 2020, Hördt et al. demonstrated that genomic differences between *Brucella* and *Ochrobactrum* were insufficient to justify separation into distinct genera [1], a finding further supported by phylogenomic analysis from Leclercq et al. [2]. As a result, species formerly classified as *Ochrobactrum* have been reclassified within the genus *Brucella*.

**Figure 1:**
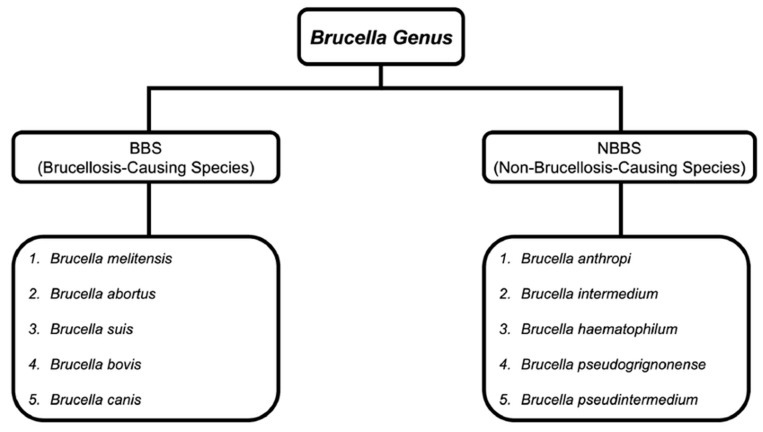
Classification of the *Brucella* genus.

Despite this reclassification, clinically meaningful distinctions remain. Classical brucellosis-causing *Brucella* species (BBS) are highly pathogenic zoonotic organisms requiring biosafety level 3 containment, whereas non-brucellosis-causing *Brucella* species (NBBS), including *Brucella anthropi*, are generally lower-risk opportunistic pathogens [3,4] (Figure 1). Notably, many clinical microbiology laboratories continue to report this organism under its former designation, reflecting ongoing debate regarding clinical nomenclature.

*Brucella anthropi* (formerly *Ochrobactrum anthropi*) was first described in 1988 and has since been increasingly recognized as a human pathogen capable of causing invasive disease [5]. Although initially considered primarily opportunistic, it is now known to cause a broad spectrum of infections, including bacteremia, osteomyelitis, and infective endocarditis [6]. We report a case of bivalvular infective endocarditis caused by *B. anthropi* and provide a focused review of previously reported cases of infective endocarditis due to this organism, with consideration of antimicrobial susceptibility patterns.

## 2. Materials and Methods

This manuscript focuses on acute endocarditis caused by *Brucella anthropi*. A narrative review of PubMed, MEDLINE, ScienceDirect and Google Scholar was conducted from database inception through 2025 using the following search terms: “*Brucella anthropi*”, “*Ochrobactrum anthropi*”, “acute endocarditis”, and “taxonomy and *Brucella*”. Peer-reviewed, English-language manuscripts were included if they reported acute endocarditis attributed to *O. anthropi* or *B. anthropi*, established by pathology, imaging, or the modified Duke criteria (2023 revision). Broader inclusion criteria were applied to account for variations in diagnostic and technological capabilities between developed and resource-limited settings.

## 3. Case Report

A 75-year-old man with a medical history of hypertension, diabetes mellitus type 2, prostate cancer treated with radiation therapy, and a prior episode of streptococcal endocarditis involving a prosthetic aortic valve (successfully treated at another institution 18 months earlier) was referred to the Infectious Disease clinic for evaluation of fever of unknown origin, weight loss, and night sweats. At the initial clinic visit, he also reported a history of three cryptogenic transient ischemic attacks.

Given his clinical history of endocarditis and his presenting symptoms, he was admitted electively to the hospital for evaluation of fever of unknown origin (FUO). In accordance with current recommendations for FUO, empiric antibiotics were withheld because he remained hemodynamically stable [7]. Blood cultures were obtained and incubated for 21 days, with subsequent subculture onto Sheep Blood Agar, Chocolate Agar, and MacConkey Agar. Organism identification was performed from growth on MacConkey Agar.

During his three-day hospitalization, he remained afebrile and clinically stable, and early culture results were negative. As he felt clinically well, he requested discharge, which was granted with instructions for close (within one week) outpatient follow-up in the Infectious Disease clinic. Approximately five days after discharge, initial blood cultures became positive for non-lactose-fermenting Gram-negative rods. After unsuccessful attempts to contact the patient, he presented to the emergency department with chest pain. The Infectious Disease team was notified, repeat blood cultures were obtained, and empiric cefepime was initiated based on the recent culture positivity.

Physical examination revealed a grade III/VI holosystolic murmur along the right parasternal border and the left second intercostal space, unchanged from prior evaluation, along with right posterior lung crackles, and a non-pruritic maculopapular rash on the torso. Laboratory studies demonstrated a leukocyte count of 6.83 × 10^3^/μL, hemoglobin of 10.4 g/dL, hematocrit of 32.7%, platelet count of 204 × 10^3^/μL, erythrocyte sedimentation rate (ESR) of 52 mm/h, and a D-dimer level of 1008 ng/mL. Urinalysis was unremarkable.

After 120 h of incubation, original blood cultures identified *Brucella anthropi*. Susceptibility testing demonstrated sensitivity to quinolones, aminoglycosides, meropenem, tetracycline, and trimethoprim-sulfamethoxazole, with resistance to cephalosporins, penicillins, and other β-lactam agents. Blood cultures obtained in the emergency room again identified *B. anthropi*, as did an additional set of blood cultures obtained prior to modification of therapy, confirming persistent bacteremia.

**Figure 2:**
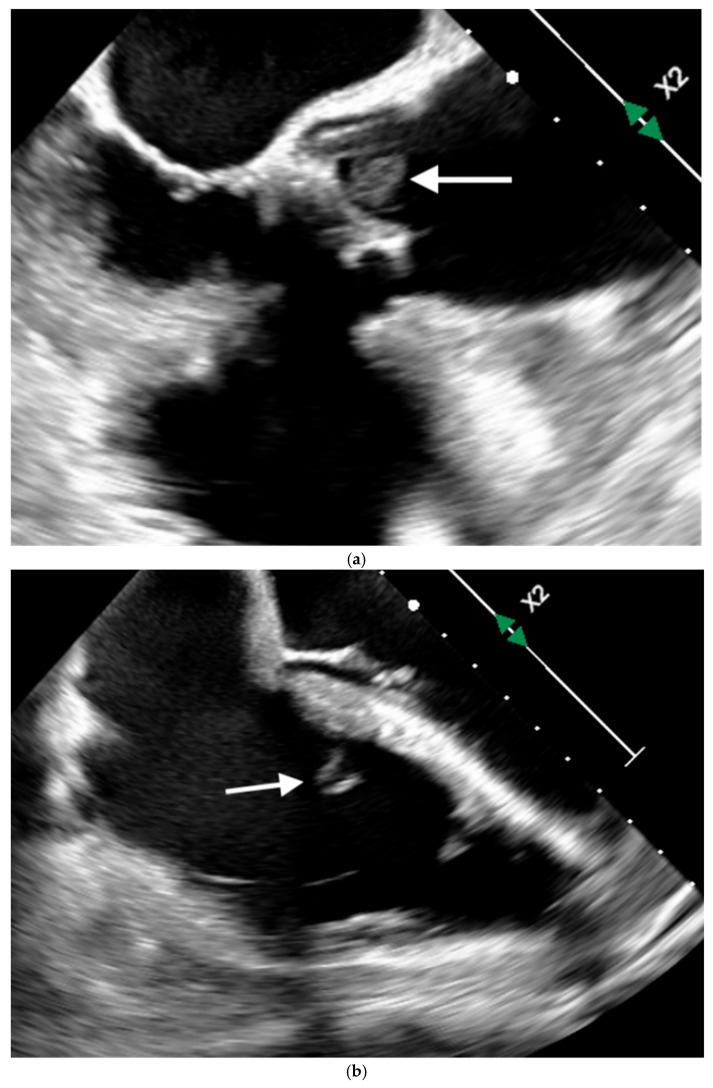
(**a**) Aortic valve vegetation (White arrow). (**b**) Tricuspid valve vegetation (White arrow).

Transthoracic echocardiography demonstrated preserved prosthetic aortic valve function without evidence of vegetations, although valve leaflet visualization was limited. Subsequent transesophageal echocardiography revealed vegetations involving both the aortic and tricuspid valves, establishing a diagnosis of acute infective endocarditis (Figure 2a,b).

Based on microbiologic and imaging findings, antimicrobial therapy was transitioned from cefepime to meropenem 1 g IV every 8 h. Due to persistent bacteremia, ciprofloxacin 250 mg orally every 12 h was added, after which blood cultures cleared.

On hospital day 8, the patient developed *Clostridioides difficile* colitis, confirmed by stool testing, and was successfully treated with oral vancomycin 125 mg every 6 h. A peripherally inserted central catheter (PICC) line was placed to facilitate completion of a six-week course of intravenous antibiotic therapy for infective endocarditis.

The patient remained clinically stable with normalization of inflammatory markers and was discharged home on hospital day 10 with close Infectious Disease follow-up. Follow-up blood cultures at 2, 4, and 6 weeks remained negative. After completing the six-week course of intravenous antibiotics, the PICC line was removed without complication.

Cardiology and cardiothoracic surgery evaluations recommended open aortic valve replacement; however, the patient died from an accidental, non-medical event a few days prior to surgery. Autopsy demonstrated chronic valvular shear damage and embolic brain lesions consistent with prior cerebrovascular events, without evidence of valve rupture or chordae tendineae injury.

## 4. Discussion

**Figure 3:**
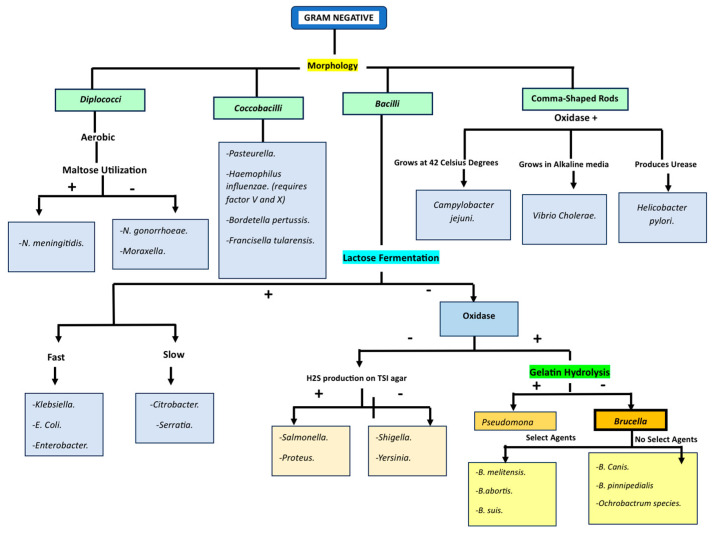
Phylogenetic placement of *Brucella anthropi*.

Advances in molecular microbiology have led to important revisions in bacterial taxonomy, with genomic analyses supporting integration of the genus *Ochrobactrum* into *Brucella* [1,2]. Despite this genetic relationship, important biological and clinical differences persist between classical brucellosis-causing *Brucella* species and *Brucella anthropi*, including differences in ecological niche, pathogenic mechanisms, and host interactions [4,8]. Recognition of these distinctions is essential for accurate interpretation of microbiologic data (Table 1 [8], Figure 3) and appropriate clinical management, particularly given the differing biosafety implications and pathogenic potential of these organisms [3,4,8].

*B. anthropi* is an environmental organism found in soil, water, and diverse ecological niches and is not known to be transmitted between humans [8]. Since its initial description and classification in the late 1980s [7], its clinical significance has become increasingly appreciated. Although originally regarded as an opportunistic or predominantly nosocomial pathogen, it has been reported in both immunocompromised and immunocompetent individuals. More than 140 cases of serious infection have been described, including pneumonia, invasive skin and soft tissue infections, bacteremia, septic arthritis, osteomyelitis, and infective endocarditis [6].

Antimicrobial susceptibility patterns of *B. anthropi* are variable and frequently characterized by resistance to β-lactam antibiotics. Resistance mechanisms are primarily mediated by plasmid-associated genes and efflux pump overexpression [9,10,11]. These features underscore the importance of susceptibility-guided therapy. In the present case, empiric cefepime was initiated based on preliminary blood culture results demonstrating non-lactose-fermenting Gram-negative rods. Following identification of *B. anthropi*, resistance to cephalosporins prompted transition to meropenem based on favorable minimum inhibitory concentration (MIC) results from our institution’s microbiology laboratory (Table 2) and side effect profile, with subsequent addition of ciprofloxacin to achieve clearance of persistent bacteremia. Notably, meropenem susceptibility has been consistently reported across prior cases [12,13,14,15,16,17,18].

The diagnostic approach in this case was consistent with established recommendations for fever of unknown origin, in which empiric antibiotics are deferred in clinically stable patients to improve diagnostic yield [8]. Prolonged incubation of blood cultures likely contributed to successful organism identification. Additionally, transesophageal echocardiography was essential in establishing the diagnosis after an initial nondiagnostic transthoracic study, highlighting the importance of advanced imaging when clinical suspicion remains high.

Review of the seven reported cases of *B. anthropi* (formerly *O. anthropi*) infective endocarditis demonstrates that infection most commonly occurs in patients with underlying valvular abnormalities or prosthetic material [12,13,14,15,16,17,18]. While some patients lacked underlying valvular predispositions, most had structural heart disease, including prosthetic valves, rheumatic heart disease, or aortic stenosis. Reported cases span a wide age range, demonstrate a male predominance, and are associated with a mortality rate approaching 25%. Only two of the eight reported cases were considered nosocomial, suggesting that *B. anthropi* endocarditis is not primarily healthcare-associated (Table 3).

To our knowledge, this is the first reported case of *B. anthropi* infective endocarditis involving both right- and left-sided valves. Prior reports have described infection limited to a single valve. Bivalvular involvement presents additional therapeutic challenges, including difficulty achieving complete eradication and a greater potential for persistent infection despite appropriate antimicrobial therapy. In our patient, the prolonged interval since prior hospitalization makes a direct healthcare-associated source less likely, although tricuspid valve involvement raises questions regarding the route of infection.

This case has limitations. Definitive molecular confirmation from excised valve tissue could not be obtained, and no clear source of infection could be established despite suspicion of prior healthcare exposure. Despite these limitations, this case provides clinically relevant insight into the diagnosis and management of a rare but increasingly recognized pathogen.

## 5. Conclusions

Ongoing advances in microbial genomics continue to refine bacterial classification, with important implications for clinical practice. Awareness of evolving nomenclature is essential to ensure accurate interpretation of microbiologic data and optimal patient care.

*Brucella anthropi* is a rare but clinically significant cause of infective endocarditis. This case documents a rare bivalvular endocarditis caused by *B. anthropi*, and highlights the importance of prolonged culture techniques, careful diagnostic evaluation, and susceptibility-guided therapy when managing infections caused by uncommon organisms.

## Figures and Tables

**Table 1:** Microbiological features that distinguish *Brucella anthropi* from brucellosis-causing *Brucella* species (BBS) [8].

*Brucella anthropi*	Brucellosis-Producing *Brucella* Species
Gram-negative rods	Gram-negative rods
Rapid growth on MacConkey agar	Slow to no growth on MacConkey agar
Mucoid morphology	Non-mucoid morphology
Non-lactose-fermenting	Non-lactose-fermenting
Catalase positive	Catalase positive
Oxidase positive	Oxidase positive
Obligate aerobes	Possibly facultative anaerobes
Motile, possessing peritrichous flagella	Non-motile, lacking flagella
Extracellular	Intracellular

**Table 2:** *Brucella anthropi* susceptibilities and MIC from our microbiological laboratory.

Antibiotic Agent	Susceptibility	MIC
Ampicillin/Sulbactam	Resistant	>16/8
Cefotaxime	Intermediate	16
Ceftazidime	Resistant	>16
Cefepime	Resistant	>16
Ciprofloxacin	Sensitive	<1
Gentamicin	Sensitive	<4
Levofloxacin	Sensitive	<2
Meropenem	Sensitive	<1
Tetracycline	Sensitive	<4
Tobramycin	Sensitive	<4
Trimethoprim/Sulfamethoxazole	Sensitive	<2/38

MIC: Minimum Inhibitory Concentration.

**Table 3:** Reported Cases of Acute Endocarditis with *Brucella anthropi* (*Ochrobactrum anthropi*).

Case	History	Clinical Presentation	Age	Sex	Definitive Treatment	Outcome	Country	Reference
1. (1990)	Bicuspid aortic valve replaced with pulmonary autograft and pulmonary homograft	1 week of lethargy, fever, malaise, pleuritic chest pain, sweating and exertional dyspnea.	28	Male	Cefuroxime 1.5 g daily + Gentamicin dose unspecified.	Complete Recovery	India	[12]
2. (2000)	Rheumatic heart disease with mild mitral stenosis, DM2, severe asthma	Sudden, severe right leg pain of 2 h duration. 15 days prior she had high-grade fever with chills and rigors. Subsequent repeated episodes of fever.	39	Female	Gentamicin 1 mg/kg IV every 8 h + Vancomycin 500 mg IV every 6 h + Ofloxacin 400 mg IV every 12 h	Complete Recovery	Pakistan	[13]
3. (2004)	HTN, rheumatic heart disease, mitral insufficiency secondary to commissurotomy, mitral valve replacement 2 years prior	3 day history of fever, abdominal pain, dyspnea.	65	Female	Meropenem 1 g IV every 6 h + Gentamicin 60 mg IV every 8 h	No complications other than gentamicin associated ototoxicity	Spain	[14]
4. (2006)	Surgery 6 months prior for traumatic rupture of bladder and terminal ileum, fracture of pelvis and pneumothorax	2 day history of fever, chills, rigors, abdominal pain, lethargy, urinary retention.	42	Male	Vancomycin 500 mg IV every 12 h + Meropenem 500 mg IV every 12 h	Death	Turkey	[15]
5. (2011)	Prosthetic aortic valve	Fever, chills	75	Male	Meropenem 500 mg IV every 8 h	Death	India	[16]
6. (2016)	History of Atrial fibrillation not on anticoagulation, coronary artery disease, moderate aortic stenosis, pacemaker for 3rd degree heart block, ESRD with failed kidney transplant, now on dialysis	2 day history of substernal sharp chest pain, nonradiating, 8/10 intensity.	58	Female	IV Meropenem dose unspecified and hemodialysis catheter removal	Complete Recovery	United States	[17]
7. (2024)	Chronic myelomonocytic leukemia transformed into acute myeloid leukemia under azacitidine. Indwelling PICC line for weekly hematological medication, with weekly blood transfusions. ICD placed secondary to dilated cardiomyopathy	Fever and mental confusion intermittently for several days.	68	Male	IV Gentamicin and Trimethoprim sulfamethoxazole unspecified dose + IV meropenem unspecified dose	Complete recovery	Italy	[18]
8. (2025)	HTN, DM2, prostate cancer treated with radiation therapy, three cryptogenic transient ischemic attacks, aortic valve replacement with bioprosthetic valve, two previous occurrences of streptococcal endocarditis	Persistent fever, malaise, chills, fatigue, significant weight loss (exceeding 20 pounds), night sweats over several weeks.	75	Male	Meropenem 1 g IV every 8 h + Ciprofloxacin 250 mg every 12 h	Complete recovery, with subsequent unrelated death	United States	Our Patient

DM2: Diabetes Type 2, HTN: Hypertension, ESRD: End Stage Renal Disease, ICD: Implantable Cardioverter-Defibrillator, PICC: Peripherally Inserted Central Line.

## Data Availability

The original contributions presented in this study are included in the article. Further inquiries can be directed to the corresponding authors.

## Abbreviations

The following abbreviations are used in this manuscript: brucellosis-causing *Brucella* species (BBS), non-brucellosis-causing *Brucella* species (NBBS), fever of unknown origin (FUO), erythrocyte sedimentation rate (ESR), peripherally inserted central catheter (PICC), minimum inhibitory concentration (MIC).
